# A Tacholess Order Tracking Method Based on Inverse Short Time Fourier Transform and Singular Value Decomposition for Bearing Fault Diagnosis

**DOI:** 10.3390/s20236924

**Published:** 2020-12-03

**Authors:** Lang Xu, Steven Chatterton, Paolo Pennacchi, Chang Liu

**Affiliations:** 1 Department of Mechanical Engineering, Politecnico di Milano, Via G. La Masa 1, 20156 Milan, Italy; steven.chatterton@polimi.it (S.C.); paolo.pennacchi@polimi.it (P.P.); 2 The State Key Laboratory of Mechanical Transmission, Chongqing University, Chongqing 400044, China; chang_liu@cqu.edu.cn

**Keywords:** tacholess order tracking, inverse short-time Fourier transform, singular value decomposition, phase extraction, bearing fault diagnosis

## Abstract

Order tracking has been widely used to diagnose failures of variable speed rotating machines. The performance of the TOT (Time-Frequency Domain Tacholess Order Tracking) methods is based on the correct separation of the target component strictly related to the shaft rotation frequency. Currently, most of the methods have focused on obtaining the instantaneous frequency with accuracy. In this paper, a new TOT method has been proposed that combines the inverse short-time Fourier transform (ISTFT) with singular value decomposition (SVD). The target component closely related to the shaft rotation frequency is selected and filtered approximately in the time-frequency domain. Hence, the ISTFT is adopted to reverse the target component into the time domain. Next, SVD is used to refine the roughly filtered target component. Finally, the phase of the refined signal is extracted to resample the original signal. The performance of the method was tested using real vibration signals collected from a large-scale test rig of a high-speed train traction system.

## 1. Introduction

Fault diagnosis of rotating machines is very important for the safe and economical operation of the machine, but in the field of fault diagnosis of rotating machines, it is easy to encounter a problem which is the variation of the rotation speed. The rotation speed variation can cause the spectral line smearing when using the spectrogram to identify the fault. It is because that the frequency indicates the repetition times per second. Order tracking has been recognized as a reliable and useful approach to alleviate or even eliminate the influence of spectral line smearing caused by varying rotational speed. If the shaft tachometer signal is available, the order tracking process can be done easily, and many significant works have been published [1,2,3]. In other situations, the tachometer signal may not be available due to space or economic limitations. In these cases, the TOT method can show its advantages.

Many interesting and useful methods have been proposed to achieve the goal of tracking orders without a tachometer signal over the past decades [4,5]. The TOT technique obtains the angle of rotation via other signals, for example, vibration signal, acoustic signal, current signal, instead of the tachometer signal [5]. Therefore, the key step of a TOT method is to separate a signal component that contains the clear information about the rotation angle of the shaft. In general, the separation method can be divided into three categories, including the time domain filtering method, the time-frequency domain filtering method, and the signal decomposition method. In the field of time domain filtering, Bonnardot et al. proposed the idea of using the gear meshing signal to perform the TOT operation [6].

Subsequently, Combet et al. proposed an automatic way to select the optimal gear meshing harmonic for TOT. But this method is only useful for low speed variation. The time-frequency filtering method has attracted the attention of many researchers. For the time-frequency filtering method, the success application of the TOT operation depends on the accurate estimation of the instantaneous frequency and the selection of the appropriate bandwidth. Zhao et al. proposed the generalized Fourier transform method, which transforms the selected harmonic into a line parallel to the time axis and uses bandpass filtering to separate the component [7]. Except for bandpass filtering, the Vold–Kalman filter has also been used to filter the selected component in the time-frequency domain [8,9,10]. Zhao et al. also proposed a time-frequency filtering method for large velocity variations based on the Chirplet-transform and the Vold–Kalman filter [11]. As for the signal decomposition method, empirical mode decomposition is often used to decompose the original signal and separate the target component for the TOT operation [12,13,14]. Chen et al. proposed a TOT method using SVD to separate the target component [15]. Some researchers have also worked on the detection of fault bases on state observers [16,17].

In this article, a new TOT method of time-frequency filtering based on ISTFT and SVD has been proposed. The target component is filtered approximately in the time-frequency domain directly. Hence, ISTFT is adopted to obtain the corresponding time domain signal of the filtered spectrum. Subsequently, SVD is used to refine or denoise the obtained time domain signal. Finally, the phase of the refined time domain signal is extracted to resample the original signal. Most of the aforementioned time-frequency filtering methods aim to get the instantaneous frequency as exactly as possible. In this paper, the processing of the time-frequency domain filtering is an approximate operation, and the refinement process is performed by SVD. Therefore, the accuracy requirement of the analysis in the time-frequency domain is not very high. Furthermore, the filtering operation of this method is performed in the time-frequency domain, which is very intuitive and easy. In summary, compared to other TOT methods of time-frequency filtering, the new method is easy to perform but still has good accuracy and reliability.

The rest of the document is organized as follows: Section 2 illustrates the fundamentals of SVD; Section 3 explores the effect of relative frequency ratio on SVD performance; Section 4 explores the effect of noise on SVD-based phase extraction; Section 5 shows the process of the new method; Section 6 tests the performance of the new method using real vibration signals.

## 2. The Fundamental of SVD

SVD has been widely recognized as a useful technique and applied in many fields, for example, principal component analysis and image compression. The SVD application object is a matrix, obtained by transforming the time series signal $x={\left[x(1),x(2),x(3),\cdots ,x(N)\right]}^{T}$ into a Hankel matrix of the size m×n space first, as follows

(1) $$A=\left[\begin{array}{ccccc} x(1) & x(2) & x(3) & \cdots & x(n)\\ x(2) & x(3) & x(4) & \cdots & x(n+1)\\ x(3) & x(4) & x(5) & \cdots & x(n+2)\\ \vdots & \vdots & \vdots & \ddots & \vdots \\ x(m) & x(m+1) & x(m+2) & \cdots & x(N) \end{array}\right]$$

where *m* = *N* − *n* + 1, *N* is the number of samples of the signal, m and n represent the number of rows and the number of columns of the Hankel matrix, respectively. Since the cases of *m* > *n* and *m* < *n* are symmetrical problems, only the case *m* < *n* will be considered.

Similar to the eigenvalue decomposition, matrix A also can be illustrated by the product of matrixes in SVD transform, as follows

(2) $$A=U\Sigma V^{\tau }$$

where orthogonal matrixes $U=[u_{1},u_{2},\ldots ,u_{m}]\in R^{m\times m}$ and $V=[v_{1},v_{2},\ldots ,v_{n}]\in R^{n\times n}$ are the left and right singular matrix respectively. Σ is a diagonal matrix with the same dimension of **A**, which the diagonal entries of Σ are non-negative values in decreasing order of magnitude, and the positive ones are the singular values of **A**. That is $\Sigma =\left[diag(\sigma_{1},\sigma_{2},\cdots ,\sigma_{m}),0\right]\in R^{m\times n}$, $\sigma_{1}\geq \sigma_{2}\geq ,\cdots ,\sigma_{m}>0$.

Matrix A can also be expressed as a summation of sub-matrices, as follows

(3) $$A=\left[u_{1},u_{2},\ldots ,u_{m}\right]\left[\begin{array}{ccccc} \sigma_{1} & 0 & \cdots & 0 & 0\\ 0 & \sigma_{2} & \cdots & 0 & 0\\ \vdots & \vdots & \ddots & \vdots & 0\\ 0 & 0 & \cdots & \sigma_{m} & 0 \end{array}\right]\left[\begin{array}{c} v_{1}^{T}\\ v_{2}^{T}\\ \vdots \\ v_{n}^{T} \end{array}\right]=\sigma_{1}u_{1}v_{1}^{T}+\sigma_{2}u_{2}v_{2}^{T}+\cdots +\sigma_{m}u_{m}v_{m}^{T}=A_{1}+A_{2}+\cdots +A_{m}$$

where A*_i_* is the corresponding sub-matrix of the *i*th sub-signal.

Time series x is decomposed and stored in *m* sub-matrices after SVD decomposition. The anti-diagonal averaging method is used to retrieve information about the time series x in each sub-matrix in a time series from the corresponding sub-matrix

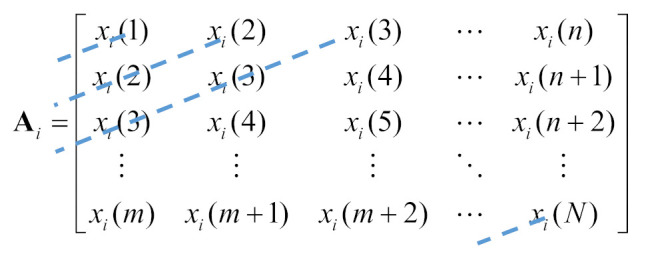
(4)

**Figure 1:**
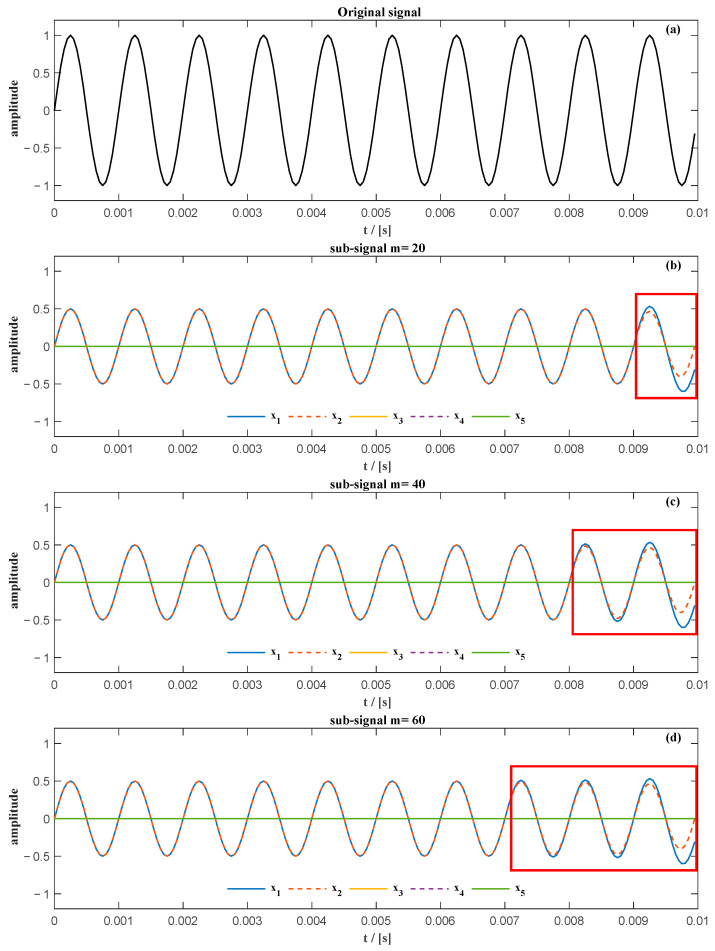
The sinusoidal signal and the first five sub-signals decomposed using the different number of rows *m*.

It is possible to show that sinusoidal signals can be conveniently decomposed into two similar sub-signals if the number of rows of the Hankel matrix is close to or greater than the number of samples in a period of it [18]. Since the gear meshing signal or shaft rotation signal is similar to the sinusoidal signal or its composition, an example is given to show the properties of SVD when applied to decompose the sinusoidal signal. For example, the expression of a generic sinusoidal signal *x*(*t*) shown in Figure 1a, is as follows

(5) $$x(t)=A\sin(2\pi f_{0}t)$$

where A=1, $f_{0}=1000$ Hz, the total number of samples of the signal is *N* = 200, the sampling frequency is 20 kHz. The number of samples in one period of the sinusoidal signal is 20. Three different number of rows *m*, namely, 20, 40, and 60, are used to decompose the sinusoidal signal. The first five sub-signals of the sinusoidal signal obtained by SVD using the different number of rows *m* are shown in Figure 1b–d. In Figure 1b–d, there is a noticeable distortion at the end of the first two sub-signals (in the red rectangle) compared with the original signal in Figure 1a and the number of distorted samples is equal to the number of rows applied to the Hankel matrix.

However, the sum of the first two secondary signals is almost the same as the original signal. Therefore, to extract the phase information of the original signal, the sum of the first two sub-signals is adopted instead of the single first sub-signal.

SVD is a linear transformation, so the phase information of the sinusoidal signal also remains in the first two sub-signals which inherit most of the information from the original signal. Phase information can be extracted through the analytical form of the signal. The analytic form of *x(t)* can be obtained from the Hilbert transform as follows

(6) $$\tilde{x}(t)=x(t)+j\cdot hilbert(x(t))$$

The Hilbert transform of *x*(*t*) is defined as

(7) $$x_{h}(t)=\frac{1}{\pi }\int_{-\infty }^{\infty }\frac{x(\tau )}{t-\tau }d\tau$$

Therefore, the instantaneous phase of the signal can be calculated as follows

(8) $$\varphi (t)=\tan^{-1}\left(\frac{hilbert(x(t))}{x(t)}\right)$$

The phase obtained using Equation (8) is bound to the interval (−π, π], therefore, the unwrapped phase is used which is a continuous function of *t* and obtained by unwrapping the instantaneous phase [19]

(9) $$\phi (t)=\varphi (t)+2\pi \cdot \left[\frac{\phi (t-1)-\varphi (t)}{2\pi }\right],\;s.t.\;-\pi \leq \phi (t)-\phi (t-1)<\pi$$

where [] obtains the closest integer.

The unwrapped phase error $\delta_{\phi }(t)$ is the phase difference between the actual unwrapped phase $\phi_{a}(t)$ and the extracted unwrapped phase $\phi_{e}(t)$, as follows

(10) $$\delta_{\phi }(t)=\phi_{a}(t)-\phi_{e}(t)$$

The percentage of the maximum phase error in one cycle is used to evaluate the influence of the unwrapped phase error on the resampling of the signal in the angular domain. The percentage of the maximum phase error in one cycle is defined as follows

(11) $$\eta =\frac{\max(\delta_{\phi }(t))}{2\pi }\times 100\%$$

**Figure 2:**
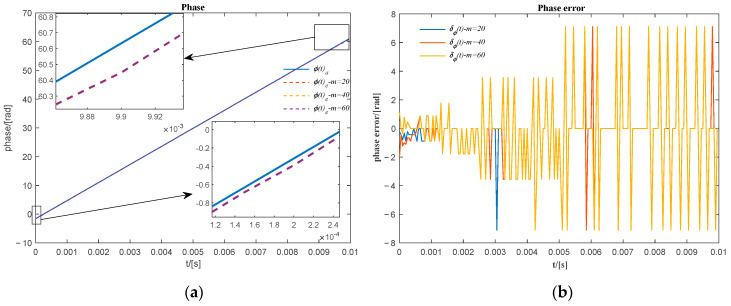
(**a**) The actual unwrapped phase and the extracted unwrapped phase using the first two sub-signals; (**b**) the phase error of the unwrapped phase extracted using the sum of the first two sub-signals.

The actual unwrapped phase of the previous sinusoidal signal and the extracted unwrapped phase of the sum of the first two sub-signals obtained from SVD using a different number of rows of the Hankel matrix are shown in Figure 2a. There is a slight difference between the actual unwrapped phase obtained from the sum of the first two sub-signals. This can also be identified by the phase error between the actual unwrapped phase and the extracted unwrapped phase shown in Figure 2b, where the phase error is very small.

## 3. Relative Instantaneous Frequency Ratio

The optimal number of rows in the Hankel matrix is only available for sinusoidal signals with a fixed oscillation frequency. If the main focus is on the shape of the sinusoidal signal, it would be better to set the number of rows *m* of the Hankel matrix near or above the optimal value to optimally separate the sinusoidal signal into two similar sub-signals. However, if the aim is to obtain the phase information of the sinusoidal signal via the sub-signals obtained from SVD, a fine selection of the number of rows of the Hankel matrix is not necessary. Since the SVD computation time is proportional to the size of the Hankel matrix, the smaller size of the Hankel matrix means less computation time. In Section 3, we will analyze the effect of the instantaneous relative frequency ratio on the phase extraction performance using the sum of the first two sub-signals obtained from SVD.

For a sinusoidal signal with variable oscillation frequency, the expression is as follows

(12) $$x(t)=A\sin(2\pi f_{0}(1+\gamma t)t)$$

where *γ* is the frequency variation rate.

The instantaneous frequency can be obtained from the time derivative of the instantaneous phase as follows

(13) $$f(t)=\frac{1}{2\pi }\frac{d\varphi (t)}{dt}=(1+2\gamma t)f_{0}$$

The relative instantaneous frequency ratio indicates the instantaneous frequency ratio between two instants of time, defined as follows

(14) $$\sigma_{12}=\frac{f(t_{2})}{f(t_{1})}=\frac{1+2\gamma t_{2}}{1+2\gamma t_{1}}$$

If the initial time $t_{1}$ is the zero-time instant, the relative instantaneous frequency ratio is $1+2\gamma t_{2}$.

### 3.1. Effect of the Relative Frequency Ratio on Phase Extraction Precision 

**Figure 3:**
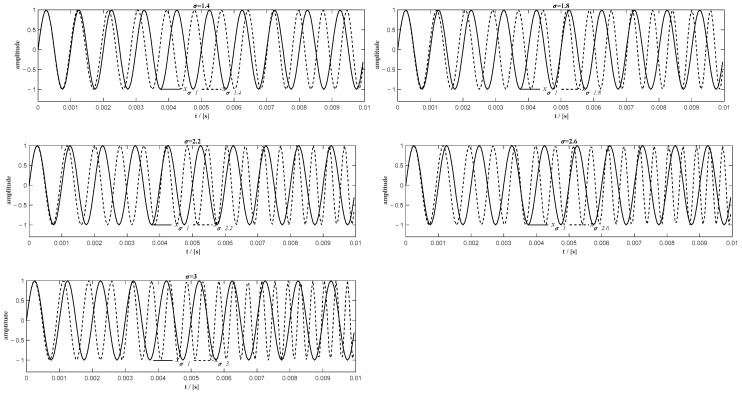
The frequency variable sinusoidal signal of the different relative frequency ratio σ.

Assuming the same parameters used for the case in Equation (5), i.e., A=1, $f_{0}=1000\mathrm{Hz}$, the sinusoidal signal variable in frequency for the values of the frequency variation rate $\gamma =\left[0,20,40,\cdots ,200\right]$, is shown in Figure 3. The corresponding relative frequency ratios $\sigma =\left[1,1.4,1.8,\cdots ,3\right]$ are obtained assuming that the initial instant of time $t_{1}$ is equal to zero.

**Figure 4:**
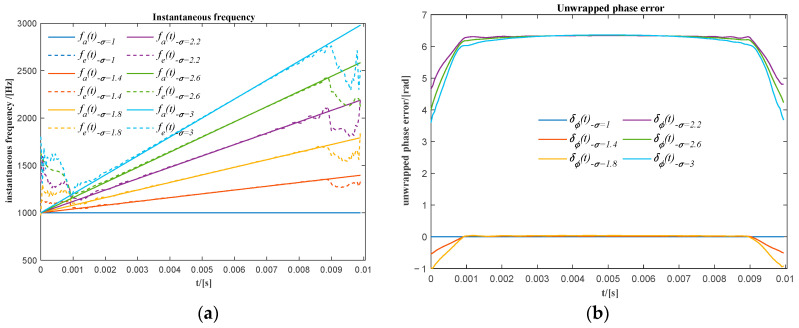
(**a**) The actual and the extracted instantaneous frequencies for different relative frequency ratios; (**b**) the phase error of the extracted unwrapped phase before phase shifting.

The optimal number of rows for the Hankel matrix required to decompose the signal into two similar sub-signals is near or greater $\frac{F_{s}}{f_{0}}=20$. The same number of rows *m* = 20 was used for the phase extraction. Then, the sum of the first two sub-signals are used to extract the phase information of the original signal. The extracted instantaneous frequency and the unwrapped phase error are shown in Figure 4. There are high and clear phase errors at the beginning and end of the signal; the number of samples with obviuos phase error is quite close to the number of rows in the Hankel matrix. According to the previous analysis, this error is mainly caused by the distortion at both ends. Therefore, the phase information at the two ends with the length of *m* is discarded. This rule will be adopted in the following analysis.

**Figure 5:**
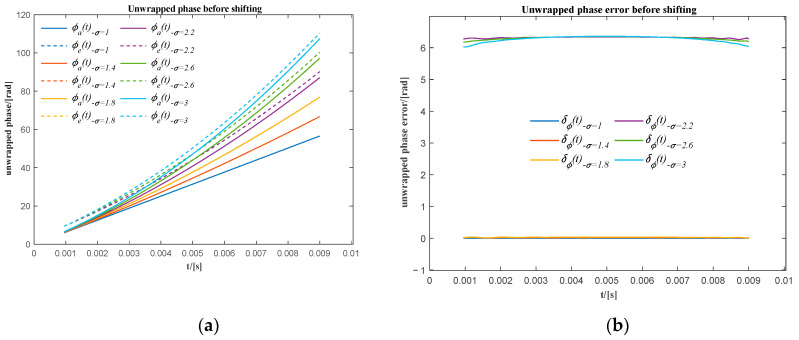
(**a**) The actual and the extracted unwrapped phase for different relative frequency ratios before phase shifting; (**b**) the phase error of the extracted unwrapped phase using the sum of the first two sub-signals before phase shifting.

**Figure 6:**
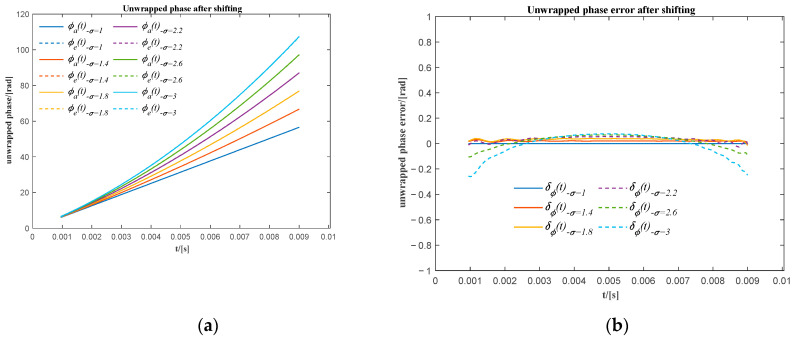
(**a**) The actual and the extracted unwrapped phase for different relative frequency ratios after phase shifting; (**b**) the phase error of the extracted unwrapped phase using the sum of the first two sub-signals after phase shifting.

In Figure 5a, when σ it is greater than 2.2, there is a phase shift of approximately 2π between the actual unwrapped phase ($\phi_{a}(t)$) and the corresponding unwrapped extracted phase ($\phi_{e}(t)$). It is also shown in Figure 5b that the unwrapped phase error ($\delta_{\phi }(t)$) oscillates around zero for σ less than 2.2, while the unwrapped phase error oscillates around 2π for σ greater than 2.2. It means there would be an overall 2π phase shift of the extracted unwrapped phase for those signals that have a high relative frequency ratio. It is caused by the distortion of the signal at the beginning of the first two sub-signals. However, the overall phase shift does not affect the precision of the resampling when using the unwrapped phase extracted in the remaining range to resample the original signal into the angular domain. After moving these unwrapped phases shifted by 2π (see Figure 6a), the maximum phase difference for all extracted unwrapped phases is approximately −0.4 rad (see Figure 6b), which means that the maximum phase error for all extracted unwrapped phase is less than 6.37% in one cycle.

**Figure 7:**
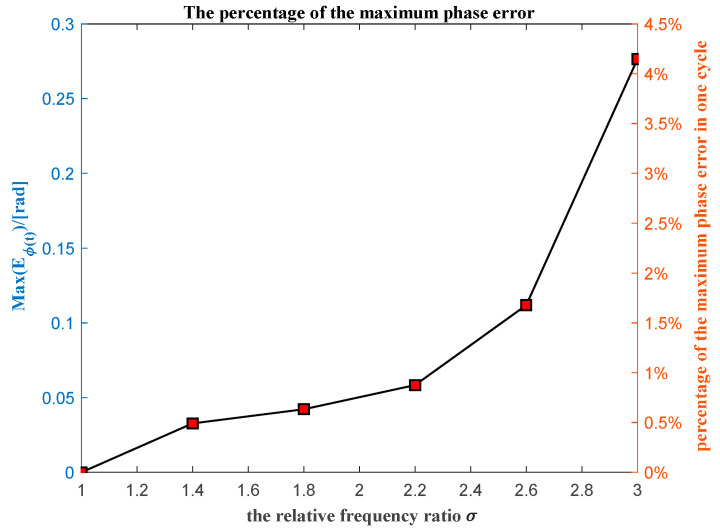
The maximum unwrapped phase error for different relative frequency ratios.

The maximum phase error carried out between the actual and unwrapped extracted phase for the different relative frequency ratio is shown in Figure 7. The maximum phase error carried out has an obvious tendency to increase along with the increase in the relative frequency ratio. It means that the maximum unwrapped phase error depends on the relative frequency ratio.

**Figure 8:**
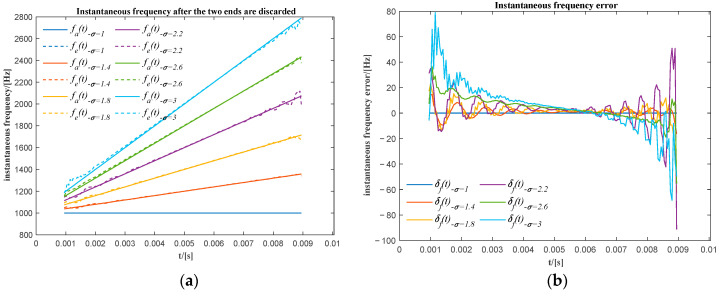
(**a**) The actual and the extracted unwrapped phase for different frequency variation ratios after the two ends are discarded; (**b**) the phase difference between the actual and the corresponding extracted unwrapped phase of the first sub-signal.

The actual instantaneous frequency ($f_{a}(t)$) and the extracted instantaneous frequency ($f_{e}(t)$) for a different relative frequency ratio after the two ends have been discarded is shown in Figure 8a. The actual instantaneous frequency ($f_{a}(t)$) and the extracted instantaneous frequency ($f_{e}(t)$) for different relative frequency ratios coincide well with each other. This can also be identified by the frequency error ($\delta_{f}(t)$) shown in Figure 8b. The instantaneous frequency error is not great especially in the mid-range.

### 3.2. The Maximum Relative Frequency Ratio for the Maximum Phase Error of Less Than 5%

The goal of phase extraction is resampling of the original signal in the angular domain using the extracted phase. Therefore, the precision of the resampling is directly related to the precision of the extracted phase. To ensure maximum phase error in a cycle of less than 5%, the relative frequency ratio σ should be limited to an appropriate range. For example, in Section 3.1, to ensure the maximum phase error in a cycle of less than 5%, the relative frequency ratio σ should be less than 3.0. In Section 3, we will analyze the effect of the starting frequency $f_{o}$ on the maximum relative frequency ratio with a maximum phase error in one cycle of less than 5%.

**Figure 9:**
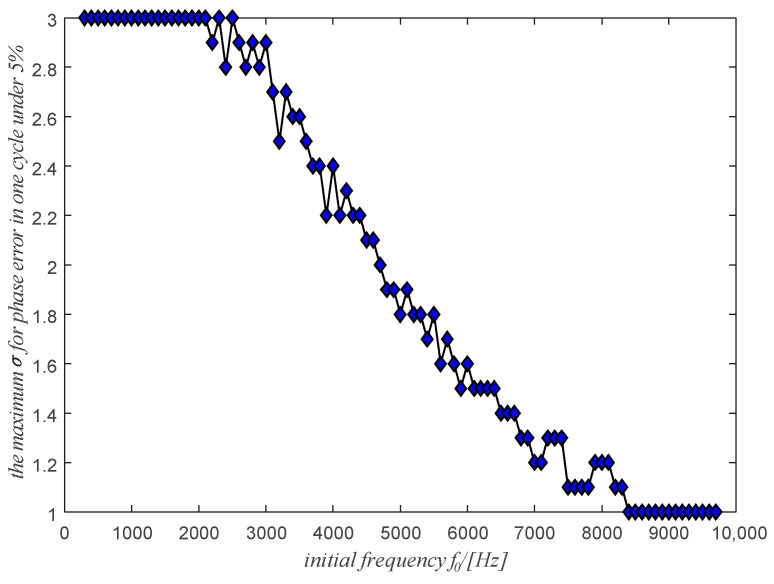
The maximum σ for the different initial frequency $f_{0}$ with the maximum phase error in one cycle under 5%.

Let’s consider the starting frequency $f_{0}$ ranging from 300 Hz to 9700 Hz and a relative frequency ratio σ between 1.0 and 3.0. The maximum value σ by which it is possible to obtain the maximum phase error of less than 5% is shown in Figure 9 as a function of the initial frequency $f_{0}$. A maximum value of σ = 3.0 is also assumed. It means that the situation of σ greater than 3.0 is not considered because in the real situation it is almost impossible to meet a case where the instantaneous relative frequency ratio is greater than 3.0. It is shown in Figure 9, to promise the phase error less than 5%, the initial frequency and the instantaneous relative frequency ratio of the signal should be in the lower region of the line in Figure 9.

## 4. The Effect of the Noise on the Phase Extraction Using SVD 

In real situations, noise is inevitably mixed with the collected vibration signal. In Section 4, the effect of noise on the phase extraction performance using the sum of the first two sub-signals obtained from SVD will be analyzed. For the sinusoidal signal in Section 2, a different level of zero-mean white Gaussian noise is added to the signal with a signal to noise ratio (SNR) between −6 dB and 6 dB. To properly decompose the sinusoidal signal into two similar sub-signals, the number of rows in the Hankel matrix should be close to or greater than $\frac{F_{s}}{f_{0}}$ = 20.

**Figure 10:**
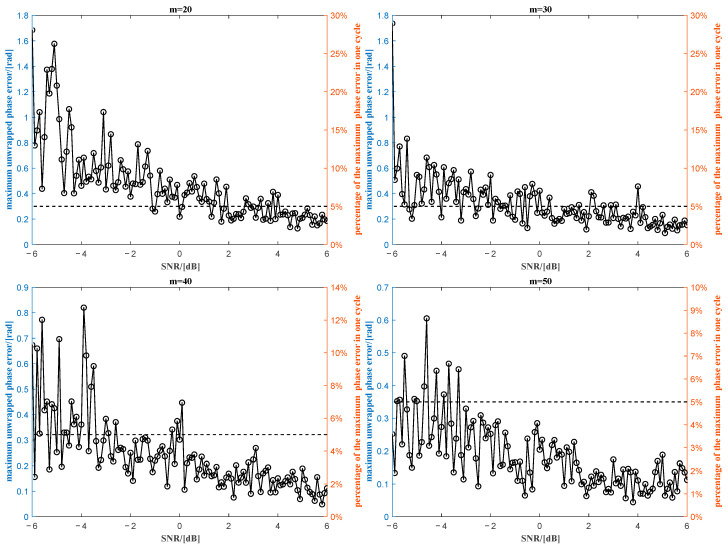
The maximum phase error of different SNRs for different number of rows of the Hankel matrix.

Four different values of the number of rows, m=[20,30,40,50], have been considered for the evaluation of the phase error shown in Figure 10. Obviously, for all four cases, the maximum phase error decreases as the SNR increases. For the number of rows m=20, to keep the maximum phase error rate in one cycle below 5% (dashed line), the SNR should be greater than approximately 4 dB. While for m=30, the general trend of the maximum phase error is quite similar to that of m=20.

However, the percentage of maximum phase error in a cycle is mostly less than 5% (dashed line) when the SNR is just above 0 dB. Furthermore, for m=50, the percentage of maximum phase error in one cycle is still less than 5% even for some SNRs close to −4 dB. It means that it is possible to increase the number of rows of the Hankel matrix to reduce the influence of noise on the phase extraction by using the sum of the first two sub-signals obtained from SVD.

## 5. A New Tacholess Order Tracking Method

**Figure 11:**
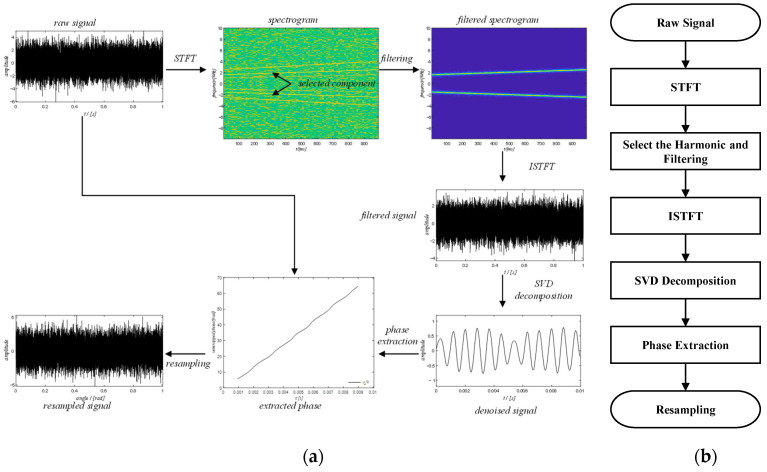
(**a**) The schematic diagram of the new method; (**b**) the flowchart of the new method.

The schematic diagram and the flow diagram of the new method are shown in Figure 11. The method mainly comprises six steps: (1) short-time Fourier transform; (2) component selection and filtering; (3) inverse short-time Fourier transform; (4) SVD decomposition; (5) phase extraction; (6) resampling. The details of the six steps will be explained below.

Step 1: Short-time Fourier transform

The spectrogram of the raw signal is obtained to analyze the components of the vibration signal and the overall trend of the variation of the component. For a time-series x(t), the time-frequency spectrum can be obtained as follows

(15) F(t,f)=STFT(x(t))

Note that the number of samples, window size and overlap size should satisfy the condition that $\frac{n_{total}-n_{overlap}}{n_{window}-n_{overlap}}$ is an integer. This is to avoid signal truncation in the following inverse short-time Fourier transform process.

Step 2: Component selection and filtering

This step allows to select the strongest harmonic of the shaft rotation frequency and to filter the selected component using an approximate bandpass filter. It is necessary to provide a rough estimate of the frequency of rotation of the shaft in an instant in advance. The selected component has a significant influence on the result of the next extraction step, so this step is very important. Here are some tips on how to select a correct harmonic of the shaft rotation frequency. The component selected must be an integer of the shaft rotation frequency or the gear meshing frequency if the system is equipped with a gearbox. Since in the previous Section 3 it is stated that the higher frequency harmonic is more sensitive to the error caused by the frequency variation, the lower frequency harmonic is selected.

**Figure 12:**
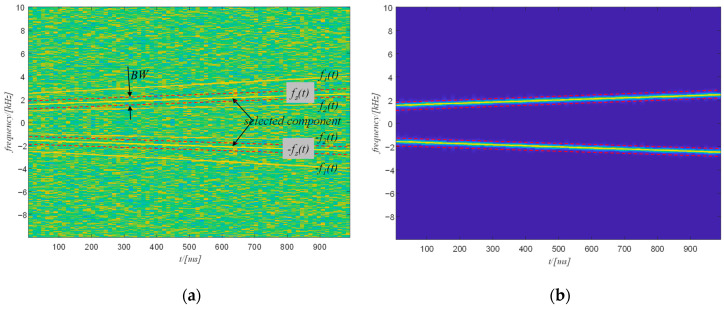
(**a**) The schematic diagram of the filtering; (**b**) the filtered spectrogram.

Generally, the selected harmonic is sandwiched by the frequency of two other components. The filtering strategy is shown in Figure 12a. The most important task of this step is to determine the bandwidth used to filter the signal in the spectrogram. Since this step is only a rough filtering, the bandwidth only needs to ensure that no frequency component relative to the other two components is included. Therefore, the bandwidth can be obtained as follows:

(16) $$BW=\min\left(\min\left(\left|f_{s}(t)-f_{1}(t)\right|\right),\min\left(\left|f_{s}(t)-f_{2}(t)\right|\right)\right)$$

where $f_{s}(t)$, $f_{1}(t)$, $f_{2}(t)$ represent the time-varying frequency of the selected component, signal component 1, and signal component 2.

The filtering process is managed in the time-frequency domain by setting to zero the amplitude of those points which are not included in the time variable frequency band, as follows

(17) $$F(t,f)=0\;\forall \;f\notin \pm \left(f_{s}(t)\pm \frac{BW}{2}\right)$$

The filtered time-frequency spectrogram is shown in Figure 12b. The selected component has been correctly separated from the raw signal. If the selected component is the component with the highest frequency, the Nyquist frequency can be used to substitute $f_{2}(t)$. Conversely, if the selected component is the component with the lowest frequency, the zero frequency can be used to replace $f_{1}(t)$.

Step 3: Inverse short-time Fourier transform

In this phase the time-frequency spectrum $F_{1}(t,f)$ is transformed into the time domain by ISTFT. Therefore, the time domain filtered signal can be obtained as follows

(18) $$x_{1}(t)=ISTFT(F_{1}(t,f))$$

Note that using the same window size and overlap size that are adopted in Step 1.

Step 4: SVD decomposition

The time domain signal $x_{1}$ obtained after ISTFT will be decomposed using SVD. To ensure the accuracy of the extracted phase, the SVD decomposition process should respect some rules according to the previous analysis. The relative frequency ratio of the filtered signal should first be evaluated by referring to Figure 9 as a function of the initial frequency and relative frequency ratio. For example, the initial frequency of the selected component shown in Figure 12b is 1550 Hz and the relative frequency ratio is $\frac{f_{s}(t=0)}{f_{s}(t=0.1)}=1.59$; this situation is in the lower zone of the 5% phase error line in Figure 9.

Therefore, the maximum phase error of the extracted phase would be less than 5% if the filtered signal can be completely decomposed at one time. However, the prerequisite is that an appropriate value of the number of rows is selected. In the previous section it was stated that increasing the number of rows in the Hankel matrix can significantly reduce the phase error. According to the conclusion in Section 4, to ensure the accuracy of the precision of the extracted phase, if the SNR of the analyzed signal is high, a relatively small value of the number of rows of the Hankel matrix can be selected, and if the SNR is low, a large number of rows in the Hankel matrix should be selected. At the same time, to ensure the phase accuracy, if the initial frequency and the relative frequency ratio of the signal is in the upper zone of Figure 9, it means that it is impossible to guarantee the phase error below 5% if the phase of the signal is extracted in one run.

Therefore, to ensure the accuracy of the extracted phase, the signal must be split into multiple segments to make sure that the initial frequency and relative frequency ratio of each segment is in the lower zone of Figure 9. So, the sum of the first two the sub-signals of each segment will be merged to extract the phase.

Step 5: Phase extraction

The time domain signal obtained from the sum of the first two sub-signals after SVD is used to extract the phase information of the original signal.

Step 6: Resampling

The extracted phase information is used to resample the original signal in the angular domain. The original signal is resampled with equal angle intervals instead of the original equal time interval. This process is done by interpolation. In this paper, the spline interpolation method is applied. Note that the total number of samples in one cycle should be no more than the number of samples in the corresponding time interval. 

## 6. Application to Experimental Data

**Figure 13:**
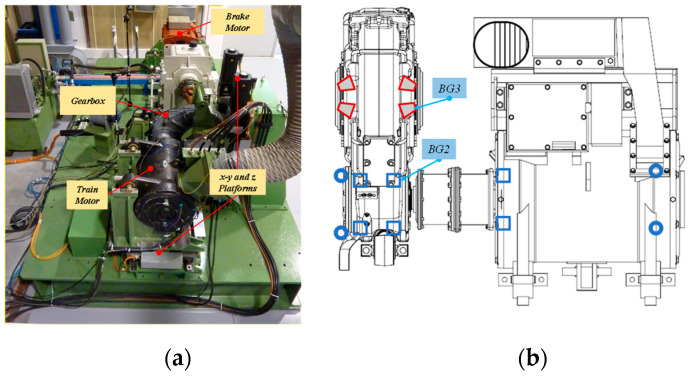
(**a**) The overall view of the test rig; (**b**) the traction motor unit.

The proposed method was validated using vibration signals collected from a large-scale test rig of a high-speed train traction system for the diagnosis of rolling elements bearings. The main components of the test stand include moving platforms, a drive motor, a gearbox, a brake motor, etc. The general view and the core of the test stand are shown in Figure 13. A tachometer is placed on the shaft for the actual shaft rotation speed. The number of gear teeth on the input and output shaft is 26 and 85 respectively. More details on the test bench can be obtained in [20]. The damaged bearings are the FAG-804989 tapered roller bearings, (labeled as BG3 in Figure 13b) located on the gearbox output shaft and the SKF NU215 (labeled as BG2 in Figure 13b), on the shaft at high speed. The bearing fault frequencies in the order domain are listed in Table 1.

**Figure 14:**
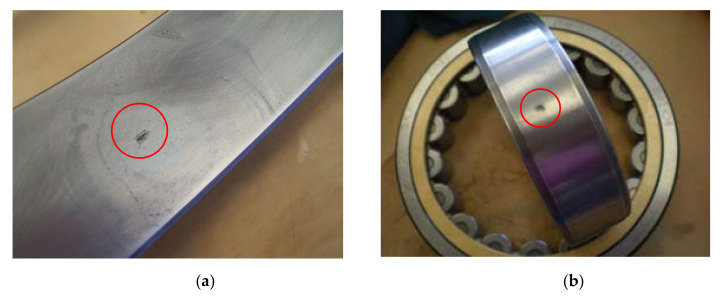
The defect of the tested bearings: (**a**) outer ring defect on FAG-804989 of case 1; (**b**) inner ring defect on SKF NU215 of case 2.

The sampling rate is 20 kHz and a 5 s signal is collected each time, but only a part of the raw signal with the sample length of $4.224\times 10^{4}$ (duration of 2.122s) is used due to the limitation of the PC memory when carrying the SVD operation. Two case studies with different operating conditions are considered to test the capability of the method applied in diagnosing bearing failures. For case study 1, an artificial spall was performed on the outer ring as shown in Figure 14a. The rotation speed of the bearing shaft varies from approximately 150 rpm to 970 rpm in 5 min. The motor runs at maximum power where the torque varies from 2200 Nm to 870 Nm. The five second signal is collected in this period. For case study 2, the defect is an artificial spall on the inner ring and the damaged inner ring is shown in Figure 14b. The rotation speed of the bearing shaft varies from about 500 rpm to 4950 rpm in 5 min. The motor operates at its maximum power where the torque varies from 2200 Nm to 540 Nm. The five second signal is collected in this period.

### 6.1. Case Study 1

**Figure 15:**
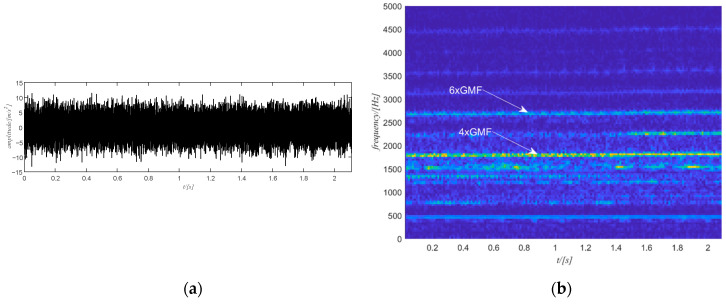
(**a**) The raw signal of case study 1; (**b**) the STFT of the raw signal.

**Figure 16:**
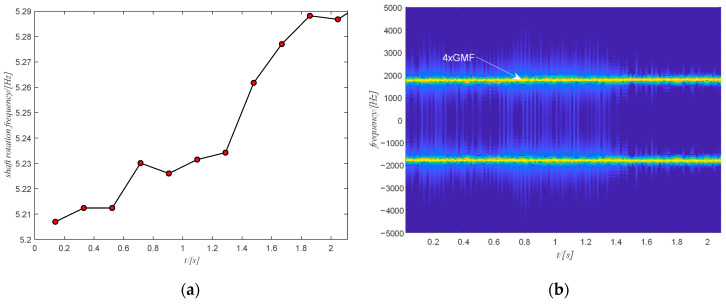
(**a**) The shaft rotation speed of case study 1; (**b**) the filtered spectrum of the selected component.

The raw vibration signal of this case study is shown in Figure 15a. The first step is to obtain the time-frequency spectrum of the signal by the STFT transform and the result is shown in Figure 15b. For the sake of clarity, only the spectrum for the positive frequency is shown in Figure 15b. Next is to select the target component. The 4th harmonic of the gear meshing frequency is selected and filtered to extract the phase information as it is stronger but also distinguishable. Note that the selection of the harmonic is performed among the five strongest components of the signal. The selection starts from the strongest one. If the strongest component is integer multiples of the gear meshing frequency, then the selection process stops. Otherwise, the selection process goes on to check the next strongest component. The filtered spectrogram is shown in Figure 16b, which includes only the 4th harmonic of the gear meshing frequency. 

**Figure 17:**
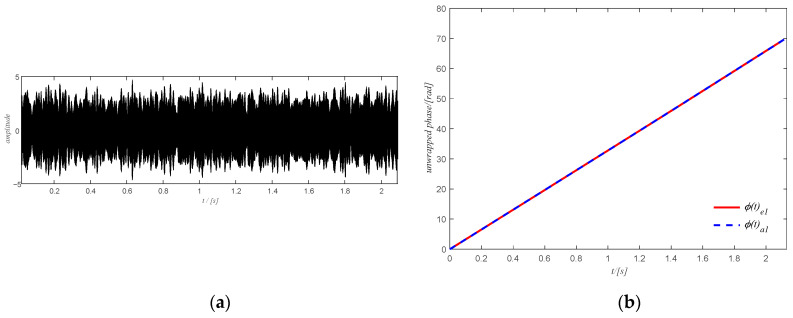
(**a**) time-domain signal of the filtered spectrum of case study 1; (**b**) final shaft phase using the time domain signal.

The estimated rotation speed of the output shaft using the tachometer signal is shown in Figure 16a where the change in the rotation speed is not very large. The corresponding time domain signal of the filtered spectrogram obtained by ISTFT is shown in Figure 17a and the extracted shaft phase using the obtained time-domain signal after SVD decomposition is shown in Figure 17b. The extracted shaft phase and the actual shaft phase are almost identical, which shows the good performance of the proposed method. The resulting shaft phase is adopted to resample the original signal. Then the resampled signal is used to detect bearing health via the square envelope spectrum (SES).

**Figure 18:**
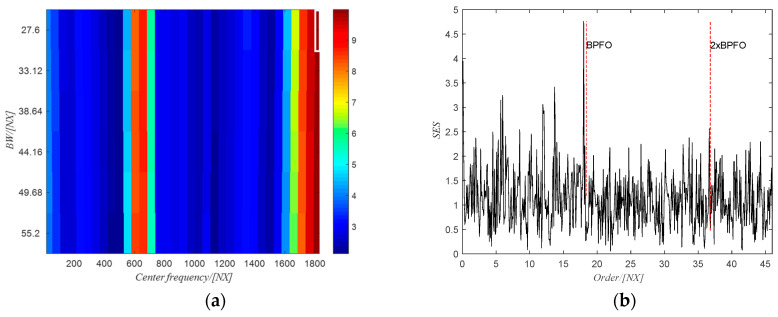
(**a**) The PMFSgram of the resampled signal; (**b**) the SES of the filtered signal.

**Figure 19:**
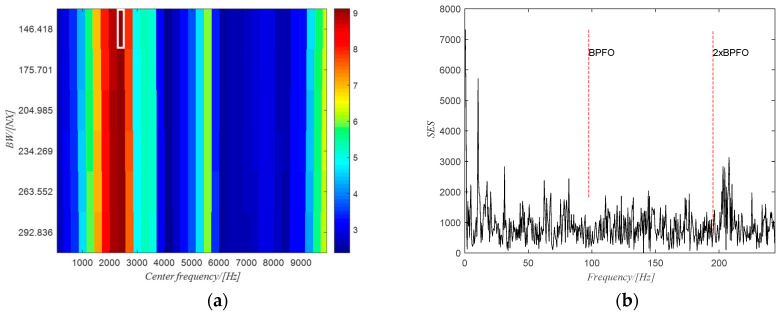
(**a**) PMFSgram of the raw signal, case 1; (**b**) SES of the filtered signal.

The PMFSgram method proposed by the same authors in [20] is adopted to obtain the optimal frequency band for the SES analysis, i.e., (1815~1843) NX, which is shown in the white rectangle in Figure 18a. The corresponding SES of the filtered signal using the obtained optimal frequency band is shown in Figure 18b. There are clear peak values at the first two harmonics of the BPFO, indicating the existence of the defect on the outer race. However, for the raw signal, the SES (see Figure 19b) of the filtered signal using the optimal frequency band (2319~2465) Hz (white rectangle in Figure 19a) obtained from PMFSgram has no peak value at the first two harmonics of the BPFO, which failed to identify the defect on the outer race.

### 6.2. Case Study 2

**Figure 20:**
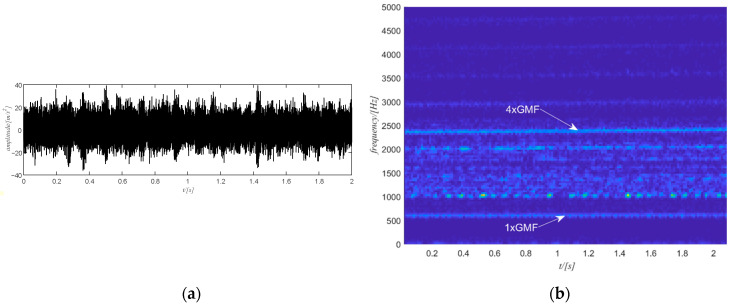
Case study 2: (**a**) raw signal; (**b**) STFT of the raw signal.

**Figure 21:**
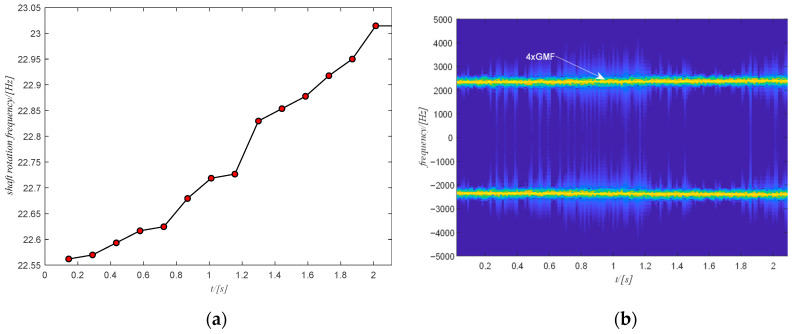
(**a**) The shaft rotation speed of case study 1; (**b**) the filtered spectrum of the selected component.

In this case study, the defect is on the bearing inner ring. The raw vibration signal (see Figure 20a) and the corresponding STFT of the signal (see Figure 20b) are shown in Figure 20. The estimated rotational speed of the output shaft is shown using the tachometer signal in Figure 21a. There are clear harmonics of the gear meshing frequency (see Figure 20b). In this case the 4th harmonic of the gear meshing frequency is selected as it is stronger and more distinguishable.

**Figure 22:**
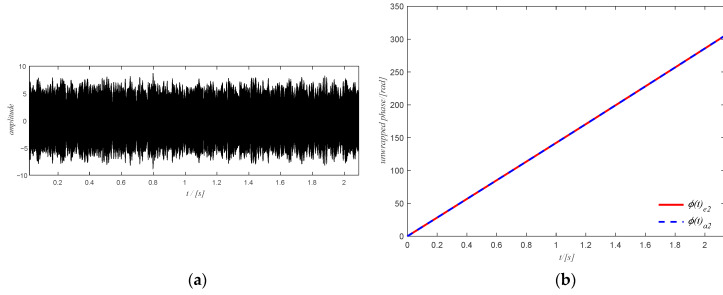
(**a**) The obtained time-domain signal of the filtered spectrum of case study 1; (**b**) the obtained shaft phase using the time domain signal.

**Figure 23:**
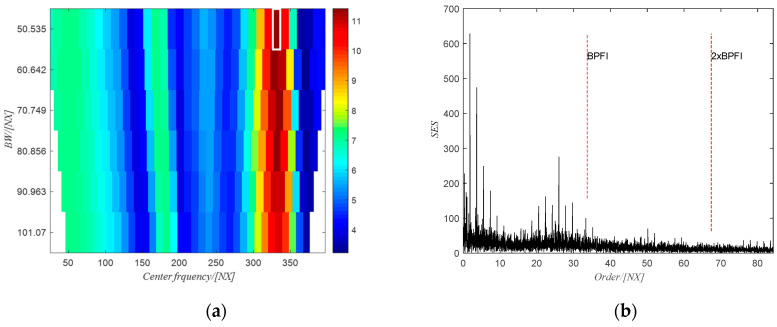
(**a**) The PMFSgram of the resampled signal; (**b**) the SES of the filtered signal.

**Figure 24:**
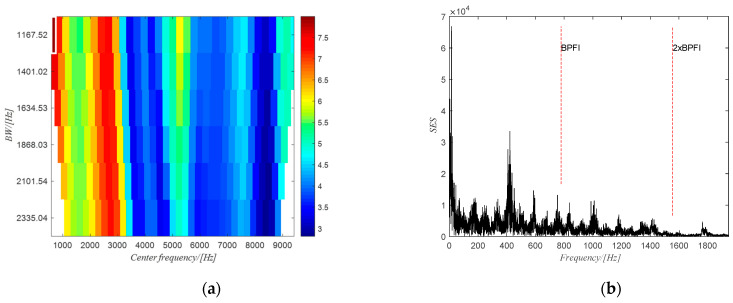
(**a**) The PMFSgram of the raw signal; (**b**) the SES of the filtered signal.

The filtered spectrogram including only the fourth harmonic of the gear meshing frequency is shown in Figure 21b. The time domain signal obtained from ISTFT of the filtered spectrogram is shown in Figure 22a and the extracted phase of the shaft using the obtained time domain signal is shown in Figure 22b. The extracted phase is quite close to the actual phase. The resulting shaft phase is applied to resample the raw signal and the resampled signal is used to identify the bearing state by means of the square envelope spectrum (SES). The optimal frequency band obtained from PMFSgram is (306~332) NX (the white rectangle in Figure 23a). The corresponding SES of the filtered signal is shown in Figure 23b. There is a peak value at BPFI, which shows the existence of the defect on the inner ring. However, for the raw signal, the optimal frequency band is (0~1168) Hz (white rectangle in Figure 24a) and the SES (see Figure 24b) of the filtered signal has no peak value in correspondence of the first two harmonics of BPFI.

Therefore, according to the analysis of the two case studies, the phase estimated using the method proposed in this work is reliable. At the same time, the estimated phase can be used to resample the raw signal and alleviate the spectral line smearing caused by the rotation speed variation. Compared to the method based on generalized modulation in reference [7], the filtering operation of the method proposed in this paper is directly in the time-frequency domain. Therefore, it is not necessary to perform the generalized Fourier transform as in reference [7]. In addition, the implementation of the adaptive STFT and generalized Fourier transform is not an easy task. Furthermore, the method in reference [7] adopts only a simple band-pass filtering before the phase extraction. Usually, the bandpass filtered signal is still very noise. The determination of the frequency band also would greatly affect the performance of the method. However, the SVD operation of the proposed method in this paper is a refinement operation to denoise the filtered component which can improve the accuracy of the extracted phase. As for the method based on the Chirplet transform and on the Vold–Kalman filter [11], the former is adopted to obtain a more precise instantaneous frequency and a constant bandwidth variable over time, while the Vold–Kalman filter is used to filter the selected component. The process is very complex and time consuming. Furthermore, this method also addresses the problem of determining the bandwidth. Therefore, the method proposed in this paper is simpler but still has acceptable performance.

## 7. Conclusions

In this paper, a new tacholess order tracking method has been proposed that combines ISTFT and SVD. STFT and ISTFT analyzes are used to filter the selected harmonic of the mesh frequency of gears or other components of the reference frequency. Hence, SVD is adopted to refine or denoise the selected component. Then, resampling is performed based on the phase information of the refined time domain signal. This method can be applied to bearing failure diagnosis to alleviate frequency smearing when rotational speed is not constant. The performance of the proposed method has been validated through case studies where vibration signals are collected from a test rig for high-speed train traction systems. The main drawback of the method is the large computational memory requirements for the SVD operation. This method will be applied in the future to monitor the condition of bearings in train traction systems.

## Figures and Tables

**Table 1:** The parameters of the tested bearings.

Bearing Code	FTF/NX	BSF/NX	BPFO/NX	BPFI/NX
SKF NU215	1.40	11.04	25.16	33.69
FAG-804989	0.47	7.43	18.40	20.60
